# Developing a Scale for the Evaluation of People With Post-prandial Distress Syndrome

**DOI:** 10.3389/fpubh.2021.695809

**Published:** 2021-06-29

**Authors:** Mengli Xiao, Jiake Ying, Yingpan Zhao, Yang Zhao, Ying Liu, Fang Lu

**Affiliations:** ^1^NMPA Key Laboratory for Clinical Research and Evaluation of Traditional Chinese Medicine, Xiyuan Hospital of China Academy of Chinese Medical Sciences, Beijing, China; ^2^National Clinical Research Center for Chinese Medicine Cardiology, Xiyuan Hospital of China Academy of Chinese Medical Sciences, Beijing, China; ^3^Department of Gastroenterology, Xiyuan Hospital of China Academy of Chinese Medical Sciences, Beijing, China; ^4^Xiyuan Hospital of China Academy of Chinese Medical Sciences, Beijing, China

**Keywords:** scale, post-prandial distress syndrome, curative effect evaluation, scale development, scale evaluation

## Abstract

**Background:** Functional dyspepsia (FD) is one of the most critical health problems worldwide. Although there has been an increased intervention to improve FD symptoms, it is difficult to compare the effect of intervention measures with the existing methods of reporting the outcome, and it is a lack of clinical evaluation tools that can be used to evaluate patients' symptoms and treatment. One way of potentially addressing this way is to offer a patient-reported symptom scoring scales, which can be self-reported by patients to highlight interventions' authenticity and reliability. Nevertheless, there is still a lack of validated patient-reported outcome instruments for post-prandial distress syndrome (PDS). This study aims to establish a symptom scoring scale to evaluate the effectiveness of interventions for PDS.

**Methods:** The study consists of two steps. The first step was to formulate the scale. Through a systematic literature review and group discussion, an item pool and scale framework were formed. Then, through the expert consultation and pre-investigation, the formal version of the scale was formed. The second step is to test the reliability and validity of the scale. The scale is tested in the target population to determine whether the reliability and validity of the scale.

**Discussion:** The improvement in patients' self-reported symptoms had a significant impact on the researchers' evaluation of the intervention's authenticity. Therefore, we develop a symptom scoring scale for reporting studies evaluating the effectiveness of PDS interventions. The scale will be used for a more significant comparison to evaluate PDS interventions' effectiveness. The scale also improves trial reporting, reducing research waste by prioritizing the collection and reporting of critical results for all relevant stakeholders.

**Clinical Trial Registration:** ChiCTR, ChiCTR2100044489. Registered on March 22, 2021.

## Introduction

Functional dyspepsia (FD) consists of a complex of symptoms, including epigastric pain or burning, post-prandial fullness, or early satiety, which cannot be found by routine examination as organic, systemic evidence, or metabolic diseases (1). And Rome IV consensus proposed to distinguish post-prandial distress syndrome (PDS; meal-related dyspeptic symptoms, characterized by post-prandial fullness and early satiation) from epigastric pain syndrome (EPS; meal-unrelated dyspeptic symptoms, characterized by epigastric pain and epigastric burning) (2). The general population's prevalence is in the range of 11.5–29.2% (3, 4). And ingestion of a meal is an essential trigger for symptom occurrence (5–7). Dyspepsia, which is the most common and representative symptom of gastrointestinal symptom, can lead to decreased quality of life and depression in patients, and studies have shown that the quality of life of patients with FD is significantly lower than that of patients undergoing gastroscopy for other reasons (8, 9). Besides, FD symptoms are prone to recurrent attacks, which seriously affect patients' quality of life and aggravate patients' economic burden. According to statistics, the UK's annual cost of indigestion is 1 billion pounds, while in the US, the average additional cost per person with FD is more than 5,000 dollars per year (10, 11).

Moreover, the symptoms of PDS often overlap with those of EPS. An epidemiological survey in Italy showed that 67.5% of patients diagnosed with FD were PDS, and 48% were EPS. As a kind of FGIDs, FD often overlaps with other disease symptoms in FGIDs because of its common physiological and pathological mechanisms. Studies have shown that patients with overlapping symptoms have a higher frequency and severity (12). Although it has some medicine can well improve patients' symptoms, it is difficult to compare the therapeutic effect of intervention measures with the existing methods of reporting the outcome, and it is a lack of clinical evaluation tools that can be used to evaluate patients' symptoms and treatment (13, 14).

The patient-reported outcome (PRO) assessment records the patient's experience of the condition in a structured form that directly states the patient's health status rather than the clinician's or anyone else's interpretation (15). Scale as a form of expression of PROs, by describing the patient can feel accepted some treatment measures after the change of the domestic symptoms of its health condition or disease, can pay more attention to themselves, highlight the actual effect of intervention effect, make the results more authenticity and reliability, is one of the commonly used methods of efficacy evaluation at home and abroad at present (16). In FGIDs clinical trials, its primary purpose is to improve all patients' signs and symptoms (2). Also, FDA proposed that for Irritable bowel syndrome (IBS), the measurement of symptoms and signs is the only existing measure that can fully determine the therapeutic effect in clinical trials (25).

Considering the higher clinical incidence and the overlap between its symptoms, we would like to develop a symptom scoring scale for PDS with overlapping symptoms of FGIDs. By searching the existing symptom scoring scales for PDS, we found still lacks the scale about PDS symptoms with overlapping symptoms in China. Therefore, we will develop a patient-filled PDS symptom scoring scale for symptom scoring to evaluate the effectiveness of therapeutic interventions for PDS. The objective is to reduce reporting bias and result variance to ensure the authenticity and reliability of clinically relevant results.

### Aim

The scale will apply to future evaluation of the effectiveness of interventions for treating patients with PDS. The scale will also be helpful for studies in PDS with overlapping symptoms of FGIDs.

## Methods and Study Design

### Study Design

**Figure 1:**
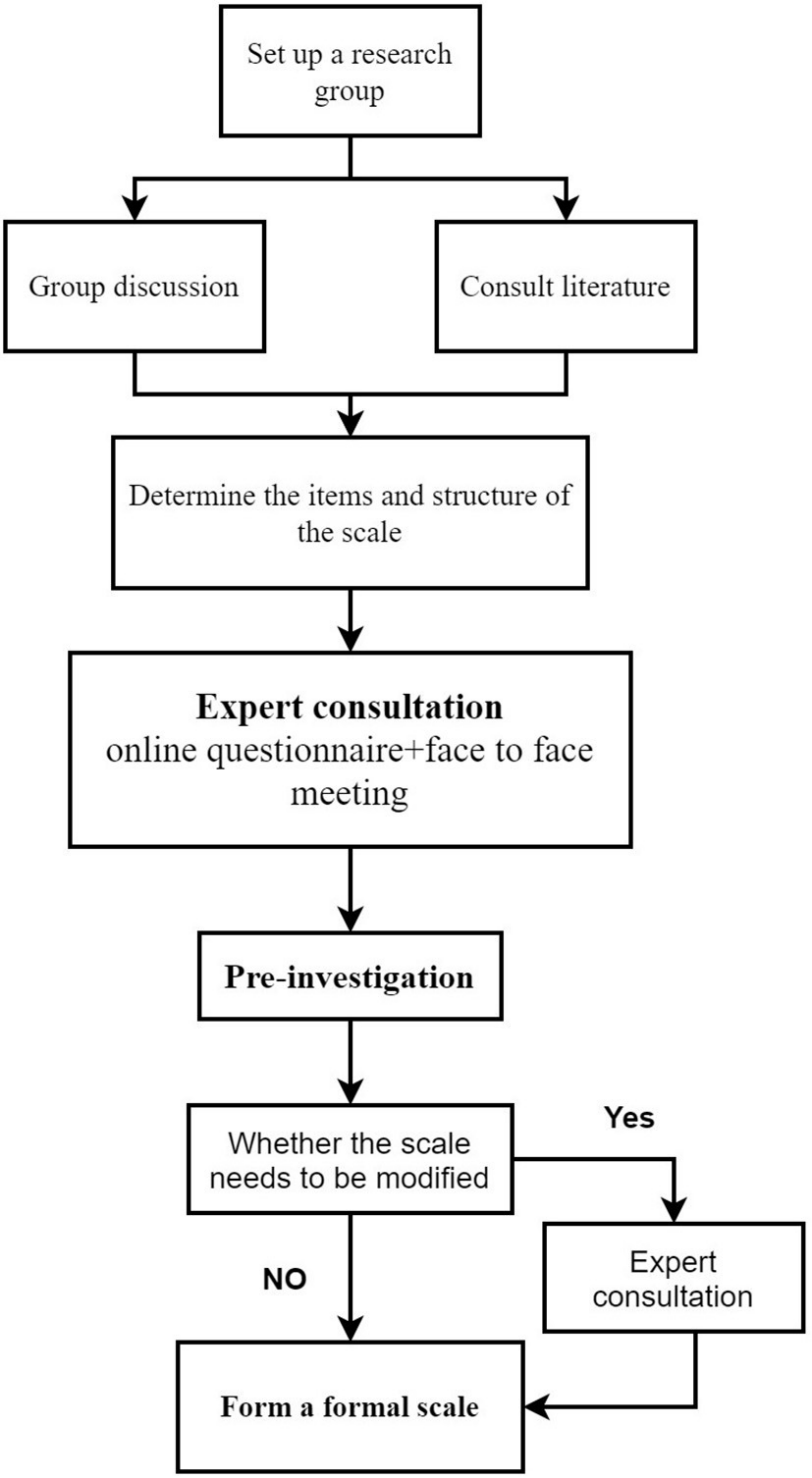
Research flow chart of developing a scale.

**Figure 2:**
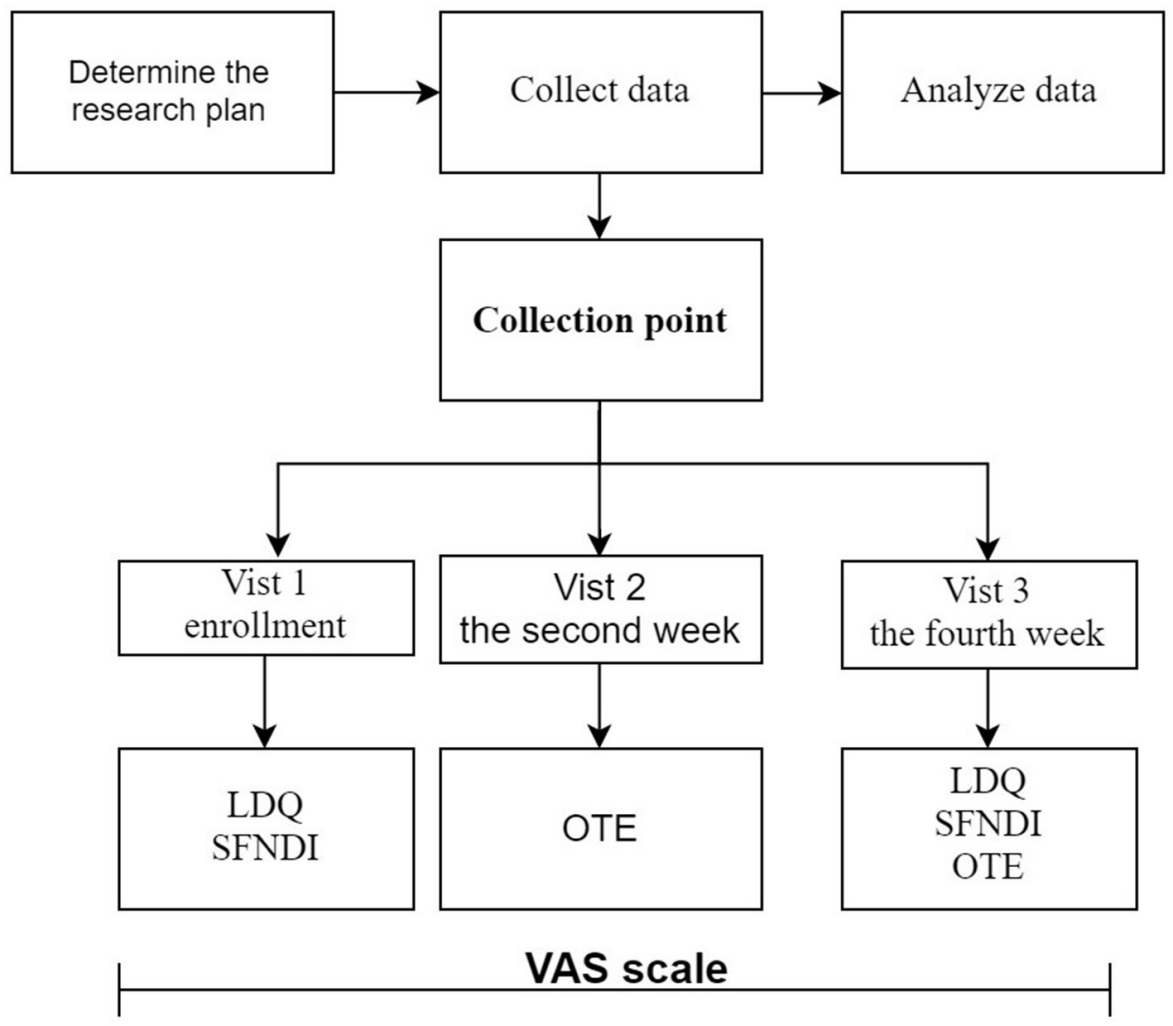
Research flow chart of the scale evaluation.

This study has two components (see Figures 1, 2):

(1) Phase 1: Develop a visual analog scoring (VAS) scale for dyspepsia symptoms: **①** Build the original entry pool and scale structure through a systematic literature review and group discussion; **②** Develop a draft of the scale through core group discussion and expert consultation; **③** Formal version of the scale was formed through investigation and evaluation of the first draft of the scale.

(2) Phase 2: Evaluate the reliability, validity, and responsiveness of the VAS scale for dyspepsia: **①** Determine the study subjects; **②** Collect the scale data; **③** Evaluate the feasibility, reliability, validity, and responsiveness of the scale.

### Methods

#### Set up a Research Group

This study has two groups. One is a core working group consisting of researchers, experts in clinical digestion, clinical evaluation methodology, and professional statisticians. The other is the scale investigation group composed of researchers and trained investigators.

#### Phase 1: Develop a VAS Scale for Dyspepsia Symptoms

**(1) Step 1:Build the original entry pool and scale structure**

By searching the book of internal medicine and digestive medicine, the scales and guidelines of PDS/FD, the existing possible symptoms of PDS were extracted, and the sorted symptoms were normalized. The core working group will pick out the most appropriate symptoms as an item.

By searching the existing scale scoring methods, we decide to use the VAS as the scale scoring method, a 10 cm line segment whose line segment scale represents the degree of symptoms. In clinical practice, a VAS is less affected by other factors in evaluating symptoms, and it can reflect the actual percentage of improvement results, which is widely used in clinical studies.

**(2) Step 2: Expert consultation**

RAND/UCLA appropriateness method (RAM) was adopted, which overcomes the disadvantages of the Delphi method in which experts do not meet so that it is difficult to reach consensus on controversial issues and participants' opinions are too dispersed in the nominal group method (17). We preliminarily consider using two rounds of expert consultations.

**①** **The first round of expert survey**

We send an e-mail to invite gastrointestinal experts with senior titles, including the vice senior and senior titles. In the e-mail, we will introduce the background, purpose, and significance of this research in simple language. We will then invite the experts to fill a questionnaire, including a 4-point Likert type scoring in rating the importance of items to the scale and suggestions on modification, supplement, and deletion.

**②** **The second round of expert survey**

The second round of experts includes gastrointestinal, methodology, and statistical. An experienced facilitator/coordinator will conduct a face-to-face meeting. At the meeting, all experts were given previous individual ratings from other experts. Each expert will give their opinions on each item's appropriateness and discuss according to the expert opinions collected last time to determine whether the items in question need to be modified, supplemented, or deleted. Before the end of the discussion, each participant will review their previous scoring results again and modify them to form the scale's first draft, and then they will evaluate the scale's draft in a 4-point Likert type scoring.

If the experts still dispute the second round of expert consultation results, the third round of evaluation would be conducted by e-mail.

**(3) Step 3: Pre-investigation**

The primary entries were filtered through core working group discussions and expert consultations. Content validity index (CVI) was used to evaluate the scale's content validity, and the final draft of the scale was formed. The scale investigation group will then pick out 30 patients with PDS from the Gastroenterology Department outpatient department and the hospital's gastroscopy room by purposed sampling. Semi-structured interviews were used to ask the respondents about the following questions: **①** past history and present history; **②** the existing symptoms and symptom severity score; **③** score the scale and explain the reasons; **④** comprehension of the question in the scale; **⑤** the processes to retrieve relevant information from memory (i.e., what does the respondent need to recall to be able to answer the question; what strategies does the respondent use to retrieve the information). After a semi-structured interview, the respondent will be invited to fill the scale.

The scale draft items were screened and modified again through pre-investigation. If the revision of the scale is involved in the pre-survey, we will consult experts again. And form a formal version of the scale.

#### Phase 2: Evaluate the VAS Scale for Dyspepsia Symptoms

##### Patient Selection

**(1) Inclusion criteria**

**①** Meet the diagnostic criteria of PDS;
**②** Age between 18 and 70 years old (including 18 and 70), male or female;
**③** Each subject is informed and voluntarily signed the informed consent form (ICF).

**(2) Exclusion criteria**

**①** Patients who cannot fill scales or record their symptoms;
**②** Pregnant or lactating females.

##### Sample Size

COSMIN recommended that the sample size should be seven times the number of items, and the sample size should be ≥100 cases (18). Assuming a dropout rate of 20%, we take 10 times the number of items. Suppose the number is <100 cases, then 100 cases are selected.

##### Observation Period

This study lasted for 4 weeks (28 ± 2 days).

##### Questionnaires

**(1) Visual analog scoring scale for dyspepsia symptoms (VAS scale)**

Based on a literature survey, the Rome IV definitions, core working group, expert consultation, and pre-investigation, a VAS scale was constructed. The scale investigated the daily symptom score of the respondents, the rating of the items is expressed as visual analog scoring (ranging from asymptomatic to unbearable) accompanied by “smiley faces,” and the weekly average of each symptom was taken as the weekly score (0–10), the higher the score was, the more severe the symptoms were.

**(2) Overall evaluation scale (OTE)**

The overall treatment efficacy is evaluated using a 7 point Likert Overall Evaluation Scale (OTE). The clinical investigators asked the subjects the following questions at the visit: “In the last week, how much have your dyspeptic symptoms been alleviated as compared to pre-treatment?” There are seven options: **①** the symptoms improved significantly, **②** the symptoms improved, **③** the symptoms improved slightly, **④** the symptoms did not change, **⑤** the symptoms aggravated slightly, **⑥** the symptoms aggravated, **⑦** the symptoms aggravated significantly. At the last visit time point of the treatment cycle, patients who selected **①**–**③** were defined as treatment responders, and those who selected **③**–**⑦** were defined as non-responders.

**(3) Leeds Dyspepsia Questionnaire (LDQ)**

The LDQ is an eight-item symptom-based questionnaire assessing dyspepsia's severity according to the frequency and the severity of various upper GI symptoms (19).

**(4) Short Form-Nepean Dyspepsia Index (SFNDI)**

The scale has 10 items that describe how stomach pain, discomfort, or other upper abdominal symptoms have affected the life over the past 2 weeks. Discomforts were rated on a 5-point Likert scale from 1 (no effect) to 5 (significant effect).

### Collection Point

The VAS scale was filled out daily by the respondents. Investigators filled out the OTE scale every 2 weeks, the time of the 2nd week and the 4th week after enrollment; And LDQ and SFNDI were completed every 4 weeks, the time of enrollment, and the 4th week of enrollment.

### Statistical Analysis

The VAS scale uses VAS to score symptoms in an asymptomatic to unbearable. These were numerically transformed into a 0–10 score range and averaged for each symptom weekly (0–10). We set up the symptoms of post-prandial fullness and early satiation, the core symptoms of PDS, as cardinal symptoms of scale and be included in the VAS diary score. The inclusion of accessory symptoms in the scale is helpful for researchers to understand the patients' epidemiology and improve the symptoms by the intervention, which is more conducive to the positioning of the intervention and the potential target population of the subjects.

**(1) Feasibility evaluation**

Actual completion and average completion time of the scale were investigated.

**(2) Construct validity**

The construct validity of the VAS scale was assessed by the content validity index (CVI). First, correct for chance agreement, which can avoid two or more experts agree on evaluating the relevance of items due to their random selection of options. CVI is composed of item-level CVI (I-CVI) and the scale-level CVI (S-CVI). I-CVI evaluates the content validity of each item, and I-CVI ≥ 0.78 is considered to have good content validity; while the S-CVI is evaluating the content validity of the entire scale, for the unanimous S-CVI (S-CVI/UA) should be no <0.8, and the average S-CVI (S-CVI/ AVE) should reach 0.90.

**(3) Criterion validity**

Criterion validity is evaluated by comparing groups with different theoretically expected VAS domain score distributions. OTE is compared with respect to post-prandial fullness and early satiation using the Mann–Whitney test. Cohen's d is also reported to quantify the degree of differentiation between groups.

**(4) Internal consistency**

The relevance of items in each VAS scale symptom domain is evaluated by the model measured at visit 2. A more direct assessment was also carried out through internal consistency measures. Cronbach's α was calculated. Generally, α values >0.6 or ideally >0.8 are sought.

**(5) Test-retest reliability**

The patient's VAS scale is filled out every day. The basic steady-state is ideal for assessing the test-retest reliability, so visit 1 and visit 2 are used to assess the test-retest reliability, and the Pearson correlation coefficient is used to measure the correlation between the two scores.

**(6) Convergent validity**

The convergent validity concept adds credibility to the VAS scale by showing that it is related to other dyspepsia symptom burden measures. The VAS scale at visit 3 was correlated with the OTE, with the SF-NDI, and with the LDQ domains corresponding to early satiation and/or post-prandial fullness. Pearson correlation coefficient is used to measure the correlation between them.

**(7) Responsiveness**

Evaluate the response degree based on the change in the respondent's VAS scale score before and after treatment.

### Data Management

All data will be completed directly by participants using an online questionnaire and stored in an online crowdsourcing platform in mainland China, which provides functions equivalent to Amazon Mechanical Turk. Data collected through the EDC will be entered directly without a paper copy. Once all data monitoring, validation, and cleanup activities have been completed, the final EDC database's output, along with any paper records, will be archived in a secure storage facility for 5 years.

### Quality Control and Assurance

A pre-specified standard operating procedure will be trained before the research, which including eligibility criteria, intervention, details in filling scale, assessment of outcomes, data management. An inspection plan will be designed for quality control. Apart from health education, patients prioritize using an online questionnaire to improve compliance. The research assistants can also track patient filling in time from the background.

## Ethics and Dissemination

### Ethical Considerations

Ethical approval for this study was granted by the Clinical Research Ethics Committee, Xiyuan Hospitals, China. This trial has been registered in ChiCTR (ChiCTR2100044489).

### Dissemination and Implementation

Upon completion of the VAS scale evaluation, the use of the VAS scale will be published and disseminated.

## Discussion

There is still a lack of available published criteria for evaluating the effectiveness of PDS interventions. By describing the changes in patients' health status or disease symptoms after receiving the intervention, the patient-reported outcome scale can pay more attention to themselves, highlight the intervention's actual effect, and make the results more authentic and reliable. By establishing a symptom scoring scale reported by patients daily, we take the average of each symptom as the weekly score, which has the advantage of prospective records and avoids the potential false recall. Also, taking the average of each symptom within a week can make the results more reliable. For the scale scoring, we adopted the VAS, which was represented by a line segment of 100 mm in length. The VAS score was less affected by other factors in evaluating symptoms, reflecting the actual percentage of improvement results. Although verbal symptom description is used in most PRO scales to describe the pattern and severity of FGID symptoms and is the outcome parameter in the PRO development process, it is not suitable for all groups of people because of the differences in the level of writing cognition and thinking ability (14, 20–23). As an alternative, pictures have great potential to promote understanding and recall of new information (24). We use the smiley face to help the patient understand the symptoms' impact on her discomfort, making the results more reliable. As the whole investigation period is as long as 4 weeks, how to prevent patients from falling out is a big problem. To solve this problem, we adopt the form of an online questionnaire survey. Researchers in the background visit and push the questionnaire, improving patient compliance and reducing unnecessary workforce and material resources. Besides, there was no pharmacological intervention for the enrolled patients, and we were unable to determine the minimal clinically important difference of the scale, which is the patients achieved the minimum improvement value. There needs further research in the future.

## Conclusion

We expect this scale to solve the two major problems of limited tools for evaluating PDS interventions' efficacy and lack of authentic and reliable tools for evaluating the efficacy. This scale can truly and reliably reflect the improvement of patients' symptoms. Because FGIDs often overlap in symptoms, the scale focuses on the main symptoms of PDS and includes the symptoms that may occur with PDS as accessory symptoms. Moreover, giving priority to collecting and reporting the possible symptoms of patients helps researchers understand their epidemiology and the improvement of each symptom by intervention measures, which is more conducive to the positioning of intervention measures and potential target subjects. Furthermore, this scale improves trial reporting, reducing research waste by prioritizing the collection and reporting of critical results for all relevant stakeholders.

## Ethics Statement

The studies involving human participants were reviewed and approved by Xiyuan Hospital, China Academic of Chinese Medical Sciences. The patients/participants provided their written informed consent to participate in this study.

## Author Contributions

FL conceived and designed the study. FL and MX wrote the protocol and manuscript, respectively. YaZ wrote the statistical analysis plan. All authors made significant contributions to the conception and design of the study protocol. All authors gave final approval of the manuscript and agreed to be accountable for all aspects of the work.

## Conflict of Interest

The authors declare that the research was conducted in the absence of any commercial or financial relationships that could be construed as a potential conflict of interest.
